# Carcinogenesis Associated with Toxin Nephropathy: Proposed Mediation by Phosphate Toxicity

**DOI:** 10.3390/cells14130952

**Published:** 2025-06-22

**Authors:** Ronald B. Brown, John G. Mielke

**Affiliations:** 1 Waterloo Institute for Complexity and Innovation, University of Waterloo, Waterloo, ON N2L 3G1, Canada; 2 School of Public Health Sciences, University of Waterloo, Waterloo, ON N2L 3G1, Canada; jgmielke@uwaterloo.ca

**Keywords:** carcinogen, nephrotoxin, toxin nephropathy, phosphate toxicity, dysregulated renal function, tumor microenvironment, glomerular filtration rate, chronic kidney disease, hyperphosphatemia, sodium phosphate cotransporter

## Abstract

Although cancer is often considered a genetic disease, genotoxic damage to nuclear DNA caused by carcinogens is not always sufficient to stimulate cancer cell growth, suggesting that other etiological factors are involved. Indeed, many carcinogens are also nephrotoxic and can impair kidney function. In turn, impaired renal function can dysregulate serum inorganic phosphate, leading to hyperphosphatemia and excess phosphate storage in tissues, which causes phosphate toxicity. Moreover, phosphate toxicity can contribute to cancer cell growth by activating cell signaling pathways, overexpressing sodium phosphate cotransporters, and stimulating excessive RNA biogenesis and protein synthesis. The present narrative review proposes a general underlying mechanism by which phosphate toxicity mediates the association of toxin nephropathy with carcinogenesis. This proposed pathway could explain why any factor that impairs renal function, including an overload of nontoxic substances, may indirectly contribute to excess phosphate sequestration in the tumor microenvironment which stimulates cancer cellular growth. Importantly, chemotherapy agents are often nephrotoxic, and carcinogenicity associated with such nephrotoxins could explain the occurrence of second tumors in treated cancer patients. More research is needed to investigate the mediating role of phosphate toxicity in the association of toxin nephropathy with carcinogenesis.

## 1. Introduction

Following the Hiroshima and Nagasaki atomic bombings in 1945, the Japanese population shunned survivors, fearing the spread of radiation poisoning and disfiguring genetic mutations in offspring [1]. Although the overall risk of cancer in survivors increased by 42%, contrary to expectations, most did not develop cancer at all. Furthermore, follow-up studies conducted over 60 years by the joint Japan and United States Radiation Effects Research Foundation [2] showed no ill effects in the offspring of survivors. The lack of ill effects thus challenges the public’s expectations of cancer outcomes associated with radiation exposure [1].

The International Agency for Research on Cancer (IARC) classifies radiation as a carcinogen according to the following definition: “a carcinogen denotes any agent, exposure to which is capable of increasing the incidence of malignant neoplasia” [3]. The reader is referred to the 2025 IARC list of carcinogenic agents classified by cancer sites with *sufficient* or *limited evidence* in humans [4]. Listing known and probable carcinogens in humans, the American Cancer Society recently confirmed that not all carcinogens cause cancer at all times [5]. In addition, although the National Cancer Institute of the U.S. National Institutes of Health (NIH) defines cancer as a genetic disease [6], evidence published by the IARC describes how direct genotoxic damage from carcinogens is not always sufficient to cause altered cell growth in cancer [7]. Indeed, damage to DNA induced by radiation or chemical agents is often used as a successful treatment to kill cancer cells [8]. Taken together, these observations suggest that additional factors are involved in the genesis of neoplasms associated with environmental exposure to carcinogenic agents.

Importantly, the lower than expected cancer rates in atomic bomb survivors serve to highlight misperceptions about carcinogens and associated cancer risks amongst the public [1]. For example, a 2022 cross-sectional online survey reported that almost half of 1494 participants (45%) agreed with the statement, “It seems like everything causes cancer” [9]. Indeed, the public’s confusion concerning carcinogens could be exacerbated by a recent update from the National Human Genome Research Institute of the NIH which reported that “over 500 substances have been identified as definitive, probable, or possible carcinogens for humans” [10]. Without doubt, the public needs greater clarity about risks associated with carcinogenicity to better inform personal decisions affecting cancer prevention. To help achieve this goal, a critical factor will be the development of a greater understanding of the biomechanisms that determine carcinogenicity.

One such biomechanism associated with carcinogenicity that deserves greater attention by researchers is nephropathy. Notably, “sufficient exposure of kidneys to ionizing radiation will cause loss of function and may lead to renal failure” [11]. Hypothetically, impaired renal function induced by radiation might contribute to carcinogenesis associated with radiation exposure. Indeed, impairment of renal function could be a mediating factor that helps explain why some people are more susceptible to the effect of carcinogenesis from radiation exposure, as shown by certain Japanese survivors of the atomic blasts. Furthermore, many carcinogens found in the environment are associated with nephropathy, acute kidney injury, and renal failure [12], and studies are urgently needed to more closely examine the association of kidney impairment with carcinogenesis.

The term “toxin nephropathy” was coined by Dr. George Schreiner in 1965 and described as:

“…any adverse functional or structural changes in the kidney due to the effect of a chemical or biological product that is inhaled, ingested, injected or otherwise absorbed, or that yields toxic metabolites with an identifiable adverse effect to the kidneys” [13].

Nephrotoxins can accumulate naturally in food, plants, mineral water, and in animal and insect venoms [12]. Many pharmaceuticals, including chemotherapy agents and immunotherapeutics used in cancer treatments, are also nephrotoxic [14]. Table 1 lists environmental nephrotoxins [15], many of which have sufficient or limited evidence for carcinogenicity according to cancer sites listed by the IARC [4]. Sources of these nephrotoxins include contaminated food and drink, consumer products, and household pesticides.

Environment and Climate Change Canada and Health Canada released a 2025 report on the state of per- and polyfluoroalkylated substances (PFAS), human-made “forever chemicals” [16], citing sufficient evidence in experimental animal studies that these substances possess both carcinogenic and nephrotoxic effects. Other research has described renal dysfunction caused by tobacco smoking [17,18] and alcohol consumption [19,20], both of which are recognized as carcinogens. Furthermore, like PFASs, alcohol and tobacco are associated with both nephrotoxicity and carcinogenicity. For example, the National Cancer Institute reported that alcohol consumption is associated with cancers of the head and neck, liver, colon and rectum, breast, and esophagus [21]. Tobacco use is associated with the same cancers, in addition to cancers of the lung, bladder, stomach, pancreas, cervix, kidney, and acute myeloid leukemia [22].

Indeed, renal dysfunction can also result from an overload of nontoxic substances, adding to the causes of toxin nephropathy defined by Dr. Schreiner. For example, a fluid overload of pure water and overhydration in people with cardiovascular disease can impair renal function [23]. Additionally, deleterious effects on renal function can be caused by an overload of essential dietary nutrients, like sodium [24] and phosphorus [25]. As the physician Paracelsus noted during the Renaissance, “Solely the dose determines that a thing is not a poison” [26].

Impaired elimination of toxins from the body due to reduced kidney function could confound laboratory carcinogenicity testing. Indeed, the IARC described how carcinogenicity testing can be confounded by chemical toxicities [27]. Moreover, writing on the challenges of assessing carcinogenicity, David G. Kaufman noted that “researchers are now confronting the difficulties of understanding the etiology and pathogenesis of multifactorial, multistep disease processes, and they are just beginning to recognize general principles that may operate in most typical cases of cancer” [28].

Kaufman’s statement implies that full elucidation of both direct and indirect carcinogenic mechanisms requires more understanding and insights into cancer etiology and cancer risk factors from a pathophysiological perspective. Still, current carcinogenicity testing appears to focus largely on direct chemical toxicities caused by external agents. Less attention is focused on indirect and mediating toxicities associated with internal metabolic reactions, including toxicities caused by metabolites—small molecular intermediates and end-products of metabolism [29]. For example, metabolite alterations are associated with arsenic carcinogenesis, yet studies are lacking “to determine how changes in metabolism are related to arsenic carcinogenesis” [30]. Recall also that Dr. Schreiner described toxin nephropathy caused by “toxic metabolites” [13]. Indeed, the present authors and others have uncovered evidence that the nontoxic metabolite inorganic phosphate (PO_4_) contributes to many biological functions within the body, yet an overload of phosphate becomes a toxic metabolite associated with cancer [31,32,33]. As a consequence of these observations, more attention needs to focus on metabolomics [29] and metabolite toxicities that contribute to carcinogenicity.

The general question investigated in the present paper is: how do renal nephropathies associated with nephrotoxicity affect tumorigenesis? Additionally, are these carcinogenic effects due to direct or indirect mechanisms, and what are potential mediating factors related to toxic metabolites? For example, epidemiological evidence linking nephropathies with malignancies indicates that acute kidney injury (AKI) is most often associated with cancers of the kidneys, liver, and bladder, as well as with multiple myeloma, lymphoma, and leukemia [34]. Yet mediating factors to explain these associations have not been fully identified. Chronic kidney disease (CKD) is also a major complication of cancer, and “the incidence of CKD in patients with cancer is higher than in the non-cancer population” [35]. Furthermore, kidney damage is an adverse effect of many cancer therapies, and has helped lead to the development of the medical branch of onconephrology [36]. However, the question remains, what are the etiological factors linking these two chronic diseases? Indeed, both cancer and CKD are associated with “poor diet” and other lifestyle factors, yet “the mechanisms behind the association between lifestyle and cancer have not been fully elucidated” [35].

One dietary factor that is a potential etiological determinant in both CKD and cancer is the previously mentioned essential mineral phosphorus [37]. Absorbed from the diet in the form of inorganic phosphate (Pi), this mineral is regulated in the serum by an endocrine network consisting of the kidneys, intestines, parathyroid glands, and bones. Furthermore, dysregulated Pi can accumulate in tissue, causing a harmful condition known as phosphate toxicity [37]. The present paper uses a pathophysiological perspective to review cellular mechanisms and other evidence linking impaired kidney function with carcinogenesis, potentially mediated by dysregulated phosphate metabolism and phosphate toxicity. The paper specifically focuses on toxin nephropathy that reduces renal function, leading directly to harmful effects of hyperphosphatemia and phosphate toxicity, and indirectly to carcinogenic effects within the tumor microenvironment.

The following section describes the method used to research and write the present paper. Section 3 of the paper discusses nephrotoxicity, including molecular mechanisms of renal Pi dysregulation. Section 4 describes five renal pathologies that are associated with cancer. The remaining sections of the paper review up-to-date cellular and molecular evidence supporting the association of Pi with cancer. Section 5 reviews the role of fibroblast growth factor receptors linking Pi and cancer. Pi cell transport and cell signaling associated with cancer are discussed in Section 6. Section 7 reviews evidence linking Pi with ribosomal RNA in the tumor microenvironment. The paper also discusses future research in Section 8.

## 2. Materials and Methods

A grounded theory literature-review method [38] was used in the present paper to rigorously and objectively review evidence from the research literature, and an explanatory theory was synthesized proposing novel associations of carcinogenicity with nephrotoxins and phosphate toxicity. Online searches using Google, Google Scholar, and PubMed used basic keywords including carcinogen, nephrotoxin, phosphate toxicity, chronic kidney disease, hyperphosphatemia, and tumorigenesis. Additional keywords in selected literature were also searched. Due to the nature of the topic being explored, the review was interdisciplinary and included findings from the fields of cellular biology, pathophysiology, epidemiology, nutrition science, and nutritional epidemiology. Indeed, an interdisciplinary approach is recommended to solve complex health problems, of which cancer is an archetype [39].

Using comparative analysis, selected findings were sorted into categories and formed into thematic relationships (direct and indirect causes and associations). In contrast to a systematic review that selects and summarizes studies by design type, no selection restrictions were placed on the type or date of findings cited in this paper. Findings were included from all sources that contributed reliable evidence supporting the development of the paper’s themes. Cited evidence included epidemiological studies (e.g., cohort, case control, cross sectional, systematic reviews and meta-analyses), as well as preclinical, clinical, in vitro, and in vivo studies. An explanatory theory began to emerge through an iterative process of data selection and induction (generalizing principles from data). Theoretical sampling was used to select additional findings from the research literature to fill in knowledge gaps. Theoretical saturation occurred when new knowledge was no longer obtained from findings in the literature, and the synthesis of a fully formed explanatory theory was complete. Evidence supporting the paper’s proposed direct and indirect relationships between nephrotoxins, carcinogenicity, and phosphate toxicity is presented as a narrative review.

## 3. Mechanisms of Nephrotoxicity

**Figure 1:**
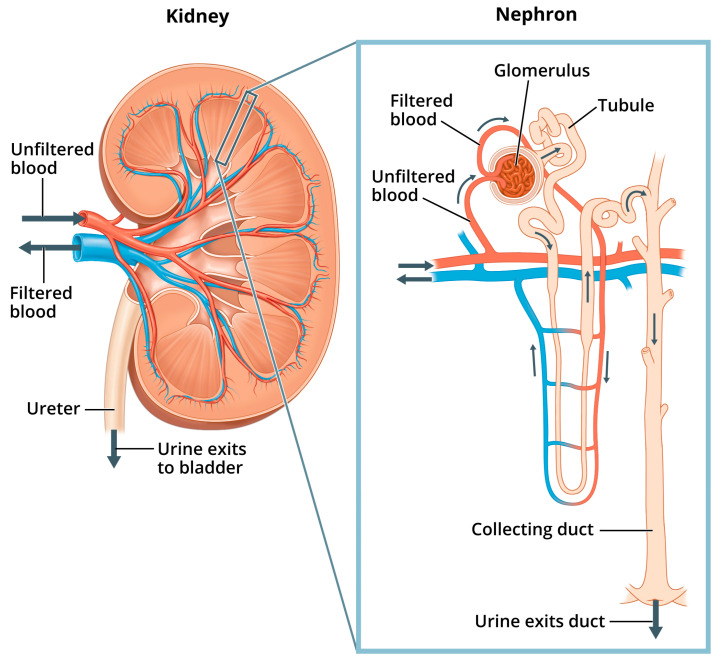
Kidney and nephron. “Image of a close up nephron and its place in the kidney. Labels on the kidney cross section show where unfiltered blood enters, filtered blood leaves, and urine exits”. National Institute of Diabetes and Digestive and Kidney Diseases, National Institutes of Health. Available online: https://www.niddk.nih.gov/news/media-library/11236 (accessed on 1 February 2025).

Renal pathophysiology induced by nephrotoxicity interferes with the kidney’s ability to fulfill its “prominent role in mediating the toxicity of numerous drugs, environmental pollutants and natural substances” [40]. Indeed, because the proportion of the cardiac output received by the kidneys is so high (25%), the kidneys are exposed to a disproportionally higher concentration of toxic substances in the blood serum compared to other tissues of the body [41]. In addition to filtration of waste from the blood, other functions of the kidneys include maintaining pH and fluid balance within the body, and hormone regulation of bone health, blood pressure, and production of red blood cells [40]. Figure 1 shows the structures of the kidney and the nephron, the kidney’s basic functional unit—each kidney contains about one million nephrons.

### Nephron Regulation of Pi

Within the nephron, “phosphate is filtered at the glomerulus, a portion is reabsorbed by the renal tubules and the excess is excreted in the urine” [42]. More specifically, serum Pi is regulated in the nephrons as the glomerular filtrate is separated from filtered blood, and excess Pi from the filtrate is excreted in the urine through the collecting ducts. Normal levels of Pi from the filtrate are also reabsorbed back into filtered blood through sodium phosphate cotransporters located mostly in the proximal tubule (the convoluted portion of the tubule in closest proximity to the glomerulus, shown in Figure 1). Additionally, hormones such as parathyroid hormone (PTH) and fibroblast growth factor 23 (FGF23), with its cofactor klotho, can reduce tubular Pi reabsorption. These hormones work by inhibiting the expression and action of sodium phosphate cotransporters, thus lowering serum Pi and increasing phosphaturia [43].

Renal function is assessed by changes in the glomerular filtration rate (GFR) as well as by levels of blood urea nitrogen (BUN), serum creatinine (sCr), and urine output [40]. Disturbances in the GFR can lower filtration of Pi from the blood and reduce the removal of excess Pi through the urine. Several pathologies are defined and diagnosed according to GFR changes [44], and Table 2 lists stages of chronic kidney disease (CKD) according to GFR.

Importantly, a GFR of <30 mL/minute in severe CKD significantly lowers Pi filtration, leading to hyperphosphatemia as Pi accumulates in the blood serum [45]. Hyperphosphatemia in stages 4 and 5 of CKD is associated with increased mortality and more rapid kidney function decline in pre-dialysis patients [46]. Kidney failure in stage 5 is also known as end stage renal disease (ESRD), which often requires hemodialysis to reduce high serum Pi levels in patients [47]. Indeed, higher intake of dietary phosphate in healthy people can also elevate levels of serum Pi [48]. Furthermore, “elevation in serum phosphate, whether episodic or chronically sustained, may trigger the secretion of regulatory hormones,” such as FGF23 and PTH, which are associated with tissue damage within the cardiovascular, renal, and skeletal systems [49]. Additionally, elevated levels of serum Pi that circulate to tissues of the body can cause phosphate toxicity, which is associated with tumorigenesis [50]. Interestingly, the association of CKD with renal cell cancer is “bidirectional and multifactorial” [51], implying that hyperphosphatemia and phosphate toxicity are potential common risk factors shared by both diseases.

Mechanisms through which hyperphosphatemia can damage renal tissue and reduce kidney function include tissue and vessel calcifications, inflammation, tubular damage, and oxidative stress. For example, general calcifications in kidney tissue, i.e., nephrocalcinosis, impair renal function through tissue deposition of calcium phosphate crystals associated with hyperphosphatemia [52]. Vascular calcification is also associated with hyperphosphatemia [53], and renal artery calcification (RAC) in patients with diabetes and proteinuria is “an independent predictor of progression to ESRD” [54]. RAC was also associated with all-cause mortality in a cohort of healthy individuals without cardiovascular disease [55]. Additionally, hyperphosphatemia in patients on maintenance hemodialysis elevated a “systemic inflammatory response” by increasing expression of the Pi transporter 1 (PiT-1) in monocytes [56] This in turn stimulated synthesis and secretion of the proinflammatory cytokines tumor necrosis factor alpha (TNF-α) and interleukin 6 (IL-6). Furthermore, phosphate overload in the kidneys produces mitochondrial dysfunction, oxidative stress, and activates autophagy of the proximal tubular cells [57]. Each of these mechanisms related to hyperphosphatemia contributes to renal tissue damage and reduced kidney function.

Importantly, compared to hyperphosphatemia, hypophosphatemia is less common in hospitalized patients (~2%) and is most likely to occur from large shifts of serum phosphate into intracellular compartments following respiratory alkalosis [58]. Hypophosphatemia is also more common in patients with cancer. However, rather than occurring from insufficient dietary phosphate intake, lower serum Pi in hypophosphatemia more often occurs in tumor genesis syndrome (TGS), as large quantities of Pi shift from the serum into rapidly growing cells of malignant neoplasms [59,60]. Dysphosphatemia, above or below normal serum levels of 2.5 to 4.5 mg/dL, is an indicator of dysregulated Pi, and even normophosphatemia does not rule out effects of phosphate toxicity in intracellular tissue [61].

## 4. Renal Pathologies and Cancer

Similar to the adverse effects of hyperphosphatemia in CKD, nephrotoxins can destroy nephrons, which can cause kidney failure [40]. Renal pathologies associated with kidney failure include dysfunction of the nephron tubules and glomerulus, kidney stone formation, polycystic kidney disease, and renal fibrosis. The following text briefly describes mechanisms of specific renal pathologies and associations with cancer, suggesting potential mediation by phosphate toxicity.

### 4.1. Renal Tubular Dysfunction

Importantly, the proximal tubule is particularly susceptible to cell loss from exposure to nephrotoxins [40], and clear cell renal carcinoma (ccRCC) in the epithelium of the proximal tubule is the most common form of kidney cancer [41]. Tubular system impairment is potentially caused by drugs and toxins that induce oxidative stress and damage the tubular mitochondria [62]. Researchers found that renal tubular dysfunction in Japanese men who were free from cadmium contamination (a nephrotoxin known to cause tubular dysfunction) was significantly associated with mortality from liver and pancreatic cancers [63]. From this association of cancer mortality with tubular dysfunction, we may infer potential mediation by hyperphosphatemia as tubular injury that eventually progresses to chronic kidney disease [64].

### 4.2. Glomerular Dysfunction

Researchers found that severe glomerular sclerosis in an animal model of chronic kidney disease was increased by higher dietary phosphate intake [65]. Glomerular dysfunction can also be caused by drugs that interfere with afferent and efferent arteriole pressure in the glomerulus, “such as nonsteroidal anti-inflammatory drugs (NSAIDs)” [62]. These drugs include angiotensin-converting enzyme inhibitors (ACEIs) and angiotensin receptor blockers (ARBs). Interestingly, NSAID use is weakly associated with reduced risk of cancer incidence, with the notable exception of a potentially increased risk of renal cancer incidence [66]. Recall that the kidneys receive a higher flow of blood with greater exposure to carcinogenic agents than other tissues [41]. Future research should explore the nephrotoxic effect of high Pi levels in the kidney due to glomerular dysfunction as a potential mediating factor linking NSAID-induced glomerular dysfunction with renal cancer.

### 4.3. Kidney Stone Formation

Recurrent kidney stone formation is linked to hyperphosphaturia [67], and researchers have suggested that “alterations in phosphorus homeostasis” may contribute to the formation of kidney stones [68], although they did not find an association with serum phosphate levels. Earlier research on kidney stones (calcium nephrolithiasis) hypothesized that a “renal phosphate leak” into the urine is due to insufficient phosphate reabsorption in the kidney causing hypophosphatemia and hyperphosphaturia [69]. But renal phosphate leak does not often occur in patients with stones, tending to refute the hypothesis. A feasible alternative explanation of a potential link between stone formation and hyperphosphaturia, albeit one that requires further investigation, is that high levels of serum Pi increase incorporation of calcium phosphate crystals into stones while releasing FGF23 and PTH, which increases phosphaturia and attempts to lower serum Pi. For example, “the majority of human kidney stones are comprised of multiple calcium oxalate monohydrate (COM) crystals encasing a calcium phosphate nucleus,” which initiates and induces stone growth [70]. Additionally, the authors of an analysis of participants in the Health Professionals Follow-Up Study suggested that higher FGF23 levels may be associated with risk for kidney stones, even though the association was on the borderline of statistical significance [68]. Kidney stones are also associated with increased risk of papillary renal cell carcinoma, which researchers suggested could be explained by common risk factors [71]. Indeed, dysregulated Pi may be a potential common risk factor mediating associations between elevated levels of FGF23, kidney stones, and renal cell carcinoma, and more research is needed in these areas.

### 4.4. Polycystic Kidney Disease

Researchers found that phosphaturia increased as levels of FGF23 and PTH rose in a murine model of polycystic kidney disease (PKD) [72], and the model showed that restriction of dietary phosphate in the mice reduced cystogenesis. The researchers also noted that contributors to decline of kidney function in PKD include “tubular injury and cystic dilation of tubules”. These findings imply a compensatory hormonal response of FGF23 and PTH to hyperphosphatemia in the murine model, and other researchers found an association of hyperphosphatemia with poor prognosis in patients with autosomal dominant PKD [73]. Patients with PKD in the National Health Insurance Research database also had a higher risk of developing renal and lung cancers [74], suggesting a link with dysregulated Pi.

### 4.5. Renal Fibrosis

Typically, renal fibrosis, particularly of the tubules and surrounding tissue, is found to be “the common final outcome of almost all progressive chronic kidney diseases” [75]. Fibrosis within the kidney is induced by chronic inflammation, which activates and expands fibroblasts to produce an extracellular matrix (scar tissue) that replaces lost renal cells [76]. In an animal model of renal fibrosis, a high phosphate diet was fed to wild-type mice for 8 to 12 weeks, which caused rapid accumulation of the extracellular matrix in interstitial tissue of the kidney when compared to mice fed a diet containing a normal amount of phosphate [77]. Notably, the renal fibrosis induced by the high-phosphate diet was attenuated by knocking out expression of the fibrotic facilitator peptidyl-prolyl isomerase, (Pin1). As a phosphorylating enzyme, future studies should investigate Pin1 upregulation in renal fibrosis and its association with a high phosphorus diet. Conversely, a restricted phosphate diet in a murine model of polycystic kidney disease inhibited the cellular pathways thought to generate the renal fibrosis seen in these animals [72]. Furthermore, renal fibrosis is associated with poor outcomes in human patients with renal cell carcinoma [78]. In addition, high levels of phosphate have also been shown to activate transforming growth factor β1 (TGF-β1) [79]. This cytokine is associated with renal fibrosis that contributes to the development of the fibrotic interstitial matrix within the tumor microenvironment during cancer metastasis.

## 5. Fibroblast Growth Factor Receptors and Pi in Cancer

As previously noted, FGF23 downregulates serum Pi by increasing urinary Pi excretion in the kidneys, and levels of FGF23 are elevated in many cancers [80]. For example, increased levels of FGF23 were found in prostate tissue of patients with prostate cancer, who also displayed severe hypophosphatemia [81]. As well, a case-control study of women with newly diagnosed breast cancer found a mean FGF23 level that was more than 2.5 times higher than in healthy women serving as controls in the study [82]. In addition, elevated FGF23 levels were observed in studies of ovarian cancer [83], colorectal cancer [84], and uterine sarcoma [85].

Each type of fibroblast growth factor (FGF) binds to a fibroblast growth factor receptor (FGFR), and abnormal activation and overexpression of FGFRs, are associated with various malignancies [86]. Recent findings have revealed an asymmetrical complex formed during FGFR binding, which could significantly influence pharmaceutical strategies to inhibit cell-signaling pathways activated by the FGFR complex [87]. One such pathway is phosphatidylinositol-3 kinase/protein kinase B (PI3K/AKT), which contributes to tumorigenesis, metastasis, and drug resistance to oncotherapies [88]. In addition to FGFR inhibitors, other cancer treatments currently under investigation include monoclonal antibodies that target FGFRs [89].

Relatedly, growth of renal cell carcinoma is claimed to depend on dysregulation of the FGF/FGF-receptor 1 (FGFR1) cell signaling pathway, yet pharmaceutical inhibition of this pathway blocks circulating levels of FGF23 and causes hyperphosphatemia [90]. These findings infer that cell-signaling by the FGFR1 pathway and circulating FGF23 contribute to compensatory responses meant to lower hyperphosphatemia. Indeed, given that phosphate overload is associated with dysregulated renal function and cancer cell growth [31,32,33], research should investigate if phosphate overload is similarly associated with dysregulation of the FGFR1 pathway in cancer.

Additionally, kinase activity of the FGFR1 is regulated, in part, by intracellular adenosine triphosphate (ATP), which binds to the tyrosine kinase domain of the transmembrane FGFR1 and triggers downstream signaling [91]. As a result, the possibility exists that elevated levels of ATP could contribute to increased kinase activity of FGFR1 in cancer cells. Indeed, evidence shows that high amounts of ATP in aggressive cancer cells are synthesized from Pi and adenosine diphosphate (ADP) by ATP-synthase within the cancer cell mitochondria during oxidative phosphorylation [92]. ATP is also produced from glycolysis of glucose in cancer cell cytoplasm by substrate-level phosphorylation [93], and glucose transporters GLUT 1 and GLUT 3 are highly expressed in cancer cells [94]. Furthermore, cancer cells also consume high levels of glutamine, and levels of Pi in cancer cells are sufficient to increase the activity of mitochondrial glutaminase which controls glutamine catabolism by cancer cells [95]. Interestingly, a rabbit model of liver cancer showed that tumoral glutaminolysis, glycolysis, and angiogenesis were inhibited, and necrosis was induced by an embolotherapy procedure that depleted the tumor of Pi using the phosphate binder sevelamer [96].

## 6. Pi Cell Transport and Cell Signaling in Cancer

The type II sodium phosphate cotransporter 2b (NaPi2b), expressed by the solute carrier family 34 member A2 gene (SLC34A2), transports Pi into cells throughout the body and is overexpressed in cancer cells. For example, SLC34A2 is overexpressed in gastric cancer stem cell-like cells, which are “regarded as the major cause of cancer recurrence” [97]; this is also the case in lung cancer stem cell-like cells from tumors in primary non-small cell lung cancer [98]. Survival of patients with colorectal cancer is also reduced [99], and colorectal cancer cell proliferation is promoted by high levels of SLC34A2 [100]. Levels of SLC34A2 in “well-differentiated endometrioid” carcinomas are overexpressed [101], and patients with tumors of the brain, pancreas, and ovaries have reduced lifespans associated with SLC34A2 overexpression [102]. Highly expressed levels of SLC34A2 in bladder cancer are associated with increased tumor size and lower patient survival, and depletion of SLC34A2 inhibits bladder tumor growth in vivo [103]. Interestingly, SLC34A2 is not overexpressed in ccRCC, and the gene’s low expression is associated with poor patient prognosis [104]. However, NaPi2b is not normally expressed in renal tissue. Instead, the kidneys express two other type II cotransporters, NaPi2a (SLC34A1) and NaPi2c (SLC34A3) [105], and studies are needed to examine the expression of these other renal type II cotransporters in association with ccRCC. Indeed, pharmacological inhibition of NaPi2a with PF-06869206 has been shown to increase phosphate excretion in rodents, which the researchers suggested could be applied therapeutically in disorders of hyperphosphatemia [106].

Researchers noted that metastasis and poor prognosis of papillary thyroid carcinoma in patients was associated with “markedly overexpressed” SLC34A2, and levels of its messenger RNA and protein in malignant thyroid tissue were elevated compared to adjacent normal tissue [107]. Additionally, phosphatase and tensin homolog/protein kinase B/forkhead box O3a (PTEN/Akt/FOXO3a) was identified by the researchers as a major signaling pathway “downstream of SLC34A2 regulated cell growth”. Coincidentally, tumorigenesis from stimulation of the Akt pathway with suppression of PTEN has been associated with high levels of dietary Pi in an animal model of lung cancer [108]. Note that kinases like Akt are enzymes that add a phosphate group to a substrate (phosphorylation), and phosphatases like PTEN are enzymes that remove a phosphate group from a substrate (dephosphorylation). Indeed, enzymatic phosphorylation and dephosphorylation of proteins regulate cell-signaling pathways, and in general, kinases are upregulated in tumorigenesis while phosphatases are suppressed [109].

Upregulation of enzymatic cell-signaling mechanisms associated with various cancers may be influenced by dysregulated levels of Pi. For example, increased expression of NaPi2b has been suggested to play a role in lung, ovarian, and breast cancers, “likely resulting from dysregulation of phosphate homeostasis” [110]. Researchers suppressed lung tumorigenesis by knocking down NaPi2b in a murine model of human lung cancer [111]. Triple-negative breast cancer cells express high levels of NaPi2b that have a strong affinity for “increasing concentrations of Pi” [112]. Interestingly, patients diagnosed with ovarian cancer with high expression of NaPi2b “continue to maintain high levels of expression over the course of their disease” [110]. This is likely related to ongoing dysregulation of phosphate homeostasis and phosphate toxicity. 

Additionally, the uptake of Pi by H+-dependent Pi transporters in breast cancer cells is five times higher than Pi uptake by Na-dependent Pi transporters [113]. This prompted researchers to suggest that H+-dependent Pi transporters could provide an alternative pathway when Na-dependent Pi transporters become saturated with Pi. The researchers also found that Pi uptake by H+-dependent Pi transporters was higher in breast cancer cells at acidic pH levels, which is consistent with the acidic tumor microenvironment [114]. Furthermore, paracellular passive diffusion of phosphate occurs between cells of the gastrointestinal tract [115]. Paracellular permeability is especially likely to occur in disease states in which tight junctions that regulate the intercellular space are compromised [116]. Future work should explore whether phosphate may enter a tumor by moving down a concentration gradient within the tumor microenvironment.

The above findings imply that more attention should focus on correcting the root cause of phosphate dysregulation in patients with cancer, including reducing perturbed renal function that may have resulted from nephrotoxicity. Indeed, nephrotoxicity is exacerbated by the known nephrotoxic effects of chemotherapy and other cancer treatments [117,118]. Evidence in the present paper associating nephrotoxins with risk of carcinogenicity could help explain why chemotherapy and radiation treatments “can put a person at higher risk for second cancers” [119]. For example, “survivors of kidney cancer have an elevated risk of development of many second primary cancers, including bladder, prostate, colorectal, lung and nervous system cancers, melanoma and non-Hodgkin lymphoma” [120]. More research is needed to investigate the mediating role of renal dysfunction and phosphate toxicity in the association of nephrotoxic cancer treatments with second cancers.

## 7. Pi, RNA, and the Tumor Microenvironment

Researchers noted that “potentially carcinogenic compounds may cause cancer through direct DNA damage or through indirect cellular or physiological effects” [121]. The researchers also noted that “studies interrogating the role of chemicals and their mixtures in dose-dependent effects on the tumor microenvironment could have important general mechanistic implications for the etiology and prevention of tumorigenesis”.

In addition to increased cancer risk from carcinogens, progression of cancer within the tumor microenvironment can increase from “an abnormal excess of limiting cell resources, including both dietary macronutrients as well as certain micronutrients” [122,123]. This includes an oversupply of the essential micronutrient phosphorus. Importantly, phosphate forms the backbone of ribonucleic acid (RNA) [124]. Therefore, as a limiting cell resource, Pi bioavailability potentially affects formation of three types of coding RNA normally involved in genetic transcription of code for protein synthesis in cell growth [125]—described in Table 3.

**Figure 2:**
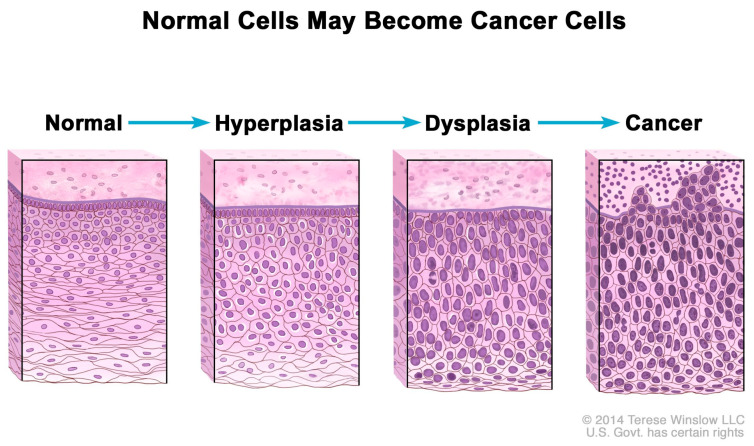
Hyperplasia and dysplasia as normal cells progress to cancer. Note the morphological changes in the cells related to increased biogenesis of rRNA. National Cancer Institute. Permission granted to use artwork. Available online: https://www.cancer.gov/publications/dictionaries/cancer-terms/def/hyperplasia (accessed on 1 February 2025).

Consequently, excess Pi sequestered in the tumor microenvironment may contribute to increases in mRNA transcription, ribosome biogenesis, and protein synthesis, processes which are normally “rate-limiting steps for cell growth and proliferation” [126]. In addition, rRNA is upregulated in cancer cells [127], and Figure 2 shows that normal cells increase in number (hyperplasia) and nuclei undergo abnormal development (dysplasia) in the progression to cancer as nuclear biogenesis of rRNA increases [128]. Indeed, experimental evidence suggests that excess Pi is incorporated into the nuclei of cancer cells during rRNA biogenesis. For example, in 1955, Ward and Griffin [129] cited their earlier experiments, in which they found that phosphorus uptake into nuclear RNA was greater in liver tumors than in normal liver, and depressed uptake of phosphorus in nuclear RNA “delayed the carcinogenic process” in precancerous rats. Excess Pi might also be associated with the observed upregulation of tRNA and protein expression in human breast cancer cells [130].

As a result, further research is needed to investigate specific cellular mechanisms by which excess Pi may upregulate formation of various types of RNA in cancer cell growth.

More general studies of the tumor microenvironment have shown that phosphate toxicity associated with sequestration of excessive phosphate, compared to normal tissue, plays an important role in cancer progression. For example, Bobko et al. demonstrated that an elevated level of interstitial Pi in the tumor microenvironment of breast cancer tissue is a biomarker of tumor progression, with Pi levels up to twice as high as in normal tissue [131]. The researchers recently found that, in contrast with other chemical parameters in the tumor microenvironment, interstitial Pi in a murine model remained consistently elevated from the earliest pre-malignancy development of cancer growth to the latest stages of cancer progression and malignancy [132]. Other researchers also found that elevated levels of Pi within the tumor microenvironment stimulated expression of the pro-angiogenic gene Forkhead box protein C2 (FOXC2) in breast cancer and lung cancer cells, which regulates neovascularization, a new source of blood vessels to the tumor, and angiogenesis, new growth within blood vessels [133]. More recently, Pi levels within the range of hyperphosphatemia were found to promote epithelial to mesenchymal transition within prostate cancer cells in vitro, which the researchers suggested supports the role of Pi in the tumor microenvironment to stimulate tumor progression [134].

## 8. Conclusions

**Figure 3:**
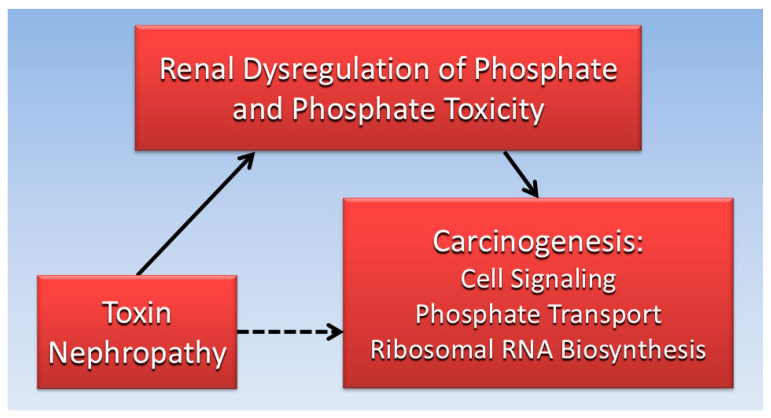
The indirect association (dotted line) of toxin nephrology with carcinogenicity is mediated (solid lines) by direct effects of renal phosphate dysregulation and phosphate toxicity.

Considered together, the evidence gathered for the current review suggests that impaired kidney function is associated with both toxin nephropathy and cancer. Furthermore, dysregulated Pi and phosphate toxicity from impaired kidney function is associated with tumorigenesis. Excess serum Pi in hyperphosphatemia can be transported into cancer cells and increase cancer cell growth by stimulating cell signaling pathways and RNA biogenesis. Ironically, nephrotoxic effects of cancer drugs and treatments may indirectly contribute to second tumors in patients. Figure 3 is a directed acyclic graph summarizing the proposed direct and indirect relationships reviewed in the present paper.

A limitation of this paper is that discussion of the renal filtrate focused mostly on Pi, with less focus on other filtrate components and metabolites that could also contribute to tumorigenesis—e.g., glucose, amino acids, urea, and electrolytes [135]. Limited space in the paper also does not allow a full discussion of mechanisms for each individual nephrotoxin, including nephrotoxic cancer drugs and treatments. Furthermore, although the grounded theory literaturereview method used in the paper increases rigor and objectivity in reviewing the research literature, data selection and analysis of literature findings and the paper’s narrative presentation are uniquely shaped by the authors’ perspectives. Strengths of the paper are the novel insights synthesized from the analysis of evidence that could lead to further research investigating dysregulated Pi and phosphate toxicity as mediating factors in the association of carcinogenicity with nephrotoxins.

More studies are needed on compounds that are both carcinogenic and nephrotoxic to determine similarities in exposure levels that generate pathological responses in both tumors and the kidneys. Future studies should investigate tumor growth promoted by phosphate toxicity in nephropathy models. For example, preclinical studies using nephropathy models, such as adenine-induced kidney injury, should test whether hyperphosphatemia accelerates tumorigenesis. Additionally, evidence that chemotherapy agents are associated with secondary tumors suggests that nephrotoxin effects of chemotherapy treatments potentially mediate this association. To help prevent secondary tumors in patients with cancer, we recommend that future studies monitor patients’ serum phosphate for rising Pi levels, and check patients’ renal function for lower GFR during cancer treatments. Changes in these indicators will allow clinicians to adjust the administration of chemotherapy agents accordingly, potentially reducing the risk of toxin nephropathy leading to phosphate toxicity and tumorigenesis.

Further research in these areas may also provide a satisfactory explanation for the prevailing public view that everything seems to cause cancer.

## Figures and Tables

**Table 1:** Environmental nephrotoxins [15] and cancer site [4].

Nephrotoxin (Cancer Site)	
Ammonia	Methyl parathion
Arsenic (kidney, urinary bladder, liver, bile duct, lung, skin, prostate)	Mercury
Aristolochic acid (renal pelvis and ureter)	Naphthalene
Barium	Ochratoxin A
Cadmium (kidney, lung, prostate)	Pentachlorophenol (lymphoma and multiple myeloma)
Carbon tetrachloride	Per- and Polyfluoroalkylated substances
Chloromethane (bile duct and lymphoma)	Thallium
Chromate and chromium (VI) (lung, nasal cavity and paranasal sinus)	Trichloroethylene (liver, bile duct, kidney, lymphoma)
Copper sulfate	Uranium (lung)
Fluoride	1,2 dibromomethane
Formaldehyde (nasal cavity and paranasal sinus, acute and chronic myeloid leukemias, other acute non-lymphocytic leukemia)	1,2 dichloromethane (lymphoma, bile duct)
Lead (stomach)	1,2 dichloropropane (bile duct)
Melamine	

**Table 2:** Stages of Chronic Kidney Disease [44].

Chronic Kidney Disease	Glomerular Filtration Rate
Stage 1—normal	>90 mL/min
Stage 2—mild	60 to 89 mL/min
Stage 3a—mild to moderate	45 to 59 mL/min
Stage 3b—moderate to severe	30 to 44 mL/min
Stage 4—severe	15 to 29 mL/min
Stage 5—failure	<15 mL/min

**Table 3:** Coding RNA [125].

Coding RNA Type	Description
Messenger RNA	“Messenger RNA (mRNA) molecules carry the coding sequences for protein synthesis and are called transcripts”.
Ribosomal RNA	“Ribosomal RNA (rRNA) molecules form the core of a cell’s ribosomes (the structures in which protein synthesis takes place)”.
Transfer RNA	‘Transfer RNA (tRNA) molecules carry amino acids to the ribosomes during protein synthesis”.

## Data Availability

No new data were created.
